# Tuning into nature: the sonic boost transforming tropical biodiversity research

**DOI:** 10.1098/rstb.2024.0044

**Published:** 2025-06-12

**Authors:** Daniela Martínez-Medina, Laurel Symes, Monica Retamosa-Izaguirre, Larissa Sayuri Moreira Sugai

**Affiliations:** ^1^ Colecciones Biológicas, Instituto de Investigación de Recursos Biológicos Alexander von Humboldt, Claustro de San Agustín, Villa de Leyva, Colombia; ^2^ K. Lisa Yang Center for Conservation Bioacoustics, Cornell Lab of Ornithology, Cornell university,; ^3^ Smithsonian Tropical Research Institute, Balboa, Ancón, Panama City, Republic of Panama; ^4^ Universidad Nacional, Heredia, Costa Rica

**Keywords:** bioacoustics, tropical ecosystems, acoustic monitoring, ecoacoustics

## 1. Introduction

Tropical ecosystems are the most biologically diverse regions on the planet, serving as natural laboratories where scientists have long posed and evaluated fundamental questions in ecology and evolution. However, these ecosystems are increasingly threatened with habitat loss, conversion and climate change, leading to unprecedented rates of biodiversity loss [1], including extinction of species that are still undescribed [2]. In response to these challenges, technological advancements have revolutionized ecological and conservation research, enhancing our ability to monitor and understand biodiversity [3]. Given the rapid pace of environmental degradation, the need for innovative and scalable monitoring approaches has never been more urgent. Acoustic monitoring has emerged as a powerful tool that expands our understanding of ecological dynamics.

Among these advancements, acoustic monitoring has gained prominence as a non-invasive and efficient tool for studying both individual species and entire ecosystems [4,5]. Initially developed for marine environments, passive acoustic techniques have since been adapted for terrestrial and freshwater ecosystems, allowing researchers to detect species' presence, analyse animal behaviour and assess ecological questions through soundscapes [6]. Animal-generated sounds provide invaluable insights into communication, social structures and ecological interactions that might otherwise be difficult to observe, particularly in the dense vegetation and towering canopies of tropical forests [7]. Beyond its applications at the species level, ecoacoustics offers a more holistic perspective by analysing entire soundscapes, providing crucial information about ecosystem integrity and environmental changes over time [8].

The integration of machine learning, combined with the advent of small, cost-effective and energy-efficient acoustic sensors, has further expanded the possibilities of bioacoustics and ecoacoustic research [9]. These innovations have enabled scientists to conduct large-scale monitoring programs and perform sophisticated analyses that push the boundaries of ecological inquiry [10–12]. Furthermore, the increasing accessibility of acoustic monitoring tools in tropical countries has encouraged broader participation from local researchers, conservation practitioners and even participatory scientists, fostering more inclusive and regionally driven conservation efforts [13]. The involvement of local communities in data collection and interpretation not only enhances conservation initiatives but also strengthens the connection between science and traditional ecological knowledge.

In this special issue, we explore the latest advances in bioacoustics and ecoacoustics in tropical regions, highlighting their applications in biodiversity assessment, conservation and ecological research. By showcasing cutting-edge studies, we aim to emphasize the crucial role of acoustics in understanding and preserving the world’s most complex and threatened ecosystems. With the continued advancement of artificial intelligence, big data approaches and the integration of acoustic monitoring with other remote sensing techniques, the future of bioacoustics and ecoacoustics holds enormous potential for revolutionizing ecological research and conservation decision-making.

## 2. The special issue

This special issue features ten articles that demonstrate the diversity of bioacoustics applications in tropical ecosystems. With contributions from more than 60 co-authors representing different countries, these studies cover a wide range of themes, including species detection, behavioural analysis, ecosystem monitoring and conservation strategies. The collaborative nature of this issue underscores the growing global interest in acoustic methods and their potential to enhance our understanding of tropical biodiversity.

The paper by wa Maina & Njoroge [14] compared different bird survey methods in the Mt. Kenya ecosystem, finding that citizen science data provided a more comprehensive species list than traditional methods. However, even with multiple approaches, rare species were still overlooked, indicating the need for further methodological refinement. Similarly, Howells *et al*. [15] assessed insect acoustic activity in the Brazilian Amazon, revealing clear daily and seasonal patterns. Their findings highlight how innovative, non-invasive techniques—such as passive acoustic monitoring (PAM)—can enhance biodiversity assessments and offer new insights into species behaviour and population dynamics.

Expanding this approach to human-modified landscapes, the study by Mendoza-Henao *et al*. [16] in the Magdalena River Valley of Colombia used PAM to examine the effects of agricultural land use on soundscape composition and diversity. The authors found that landscape characteristics, such as patch shape and the proportion of natural cover, strongly influenced acoustic activity and soundscape homogenization. These results underscore the value of integrating PAM with landscape metrics to inform sustainable land management and promote ecological resilience in agricultural settings.

Acoustic monitoring also provides valuable insights into species-specific behaviour in relation to environmental and anthropogenic pressures. In the Congo Basin, research on the African forest elephant (*Loxodonta cyclotis*) by Verahrami *et al*. [17] used PAM to evaluate responses to gun hunting in and around Nouabalé-Ndoki National Park. Results showed a measurable decline in elephant occupancy and an increase in nighttime vocal activity following gunfire events—behavioural shifts that may have long-term implications for foraging, reproduction and forest ecosystem dynamics. This work highlights PAM’s utility in studying elusive species in dense tropical forests and reinforces its importance in conservation monitoring.

At a community level, bird vocalization patterns in the Western Ghats of India were analysed by Ramesh *et al*. [18], revealing that vocal activity was significantly raised at dawn across species. The study found that territoriality and diet, rather than environmental factors, were the main drivers of dawn-biased vocal behaviour. These findings contribute to a growing body of literature exploring the social and ecological determinants of acoustic behaviour in tropical birds, and they demonstrate the value of multi-taxon PAM studies in uncovering patterns at the community scale.

Machine learning is increasingly being used to enhance PAM for ecological assessments. The study of Williams *et al*. [19] on reef soundscapes demonstrated that pretraining models with diverse datasets, including bird audio, significantly improves classification accuracy. Similarly, Barroso *et al*. [20] applied artificial intelligence to classify fish sounds in rocky reefs, achieving high accuracy and identifying key acoustic features. These advancements highlight the potential of AI to automate and refine species identification, reducing the need for extensive manual annotation and computational costs.

Acoustic monitoring has also proven effective for studying the behavioural patterns of species vulnerable to climate change. Research by Chirino *et al*. [21] on the critically endangered lemur leaf frog (*Agalychnis lemur*) used automated signal detection to analyse calling activity, revealing that temperature and humidity significantly influence vocal behaviour. The study by Nguyen Chi *et al*. [22] on the crested argus (*Rheinardia ocellata*) in Vietnam utilized PAM and machine learning to detect calls, identify environmental predictors of vocalization and track seasonal variations. These findings underscore the importance of sound-based monitoring for understanding species responses to environmental changes.

Additionally, insect-generated soundscapes provide valuable insights into ecosystem health and biodiversity. Despite their ecological significance, insects have been underrepresented in acoustic biodiversity monitoring. A review by Riede & Balakrishnan [23] emphasized the need to integrate PAM into conservation strategies, proposing a roadmap for leveraging artificial intelligence and citizen science to enhance data analysis. Overall, these studies collectively demonstrate the power of acoustic monitoring and machine learning in biodiversity research, offering innovative approaches to conservation and species management in the face of global environmental challenges.

## 3. Challenges and horizons

The research featured in this issue highlights some of the ongoing challenges for bioacoustics, as well as the potential for future horizons and contributions of the approach. Access to equipment and expertise remain a substantial challenge in many parts of the world, as for many tropical areas, but high-quality audio and acoustic technologies are now available in many regions. Developing and sharing open-source recording equipment designs could reduce many of the challenges and costs associated with importing, repairing and maintaining recording equipment. In addition to hardware issues, access to software and personal expertise still remains a challenge. Many practitioners, especially newcomers, may not have direct personal contact with others who use the approach, meaning that hardware, software, data storage and analysis challenges may be faced in isolation, with higher likelihood that obstacles more easily lead projects to be terminated. However, the growing accessibility of virtual materials and the emergence of social networking around particular research subjects have an increasing role in connecting practitioners, especially from the Global South.

Although bioacoustics enable us to draw critical ecological information from the presence of species, deriving estimates based on abundance and density remains labour-intensive and costly. Both hardware and software advances may make these goals more feasible, through techniques such as multi-microphone arrays, multi-element source separation and identification of individual vocal signatures using machine learning approaches.

With the consolidation of standards in acoustic monitoring and datasets being collected across tropical regions, the potential of synthesis can help address theoretical and applied questions in ecology and conservation. Synthesis of data across sites and regions will unlock insights about habitat requirements, population changes, geographic variation and impacts of management that transcend individual locations. Effectively summarizing data across sites will require substantial coordination and infrastructure development. However, if implemented successfully, cross-project synthesis offers a particularly rich opportunity to generate broadscale and comparative insights that can be used in conservation efforts at the regional and local levels.

Bioacoustics is a cross-disciplinary field applicable to multiple taxonomic groups, enabling us to address ecological, behavioural and taxonomic questions. This versatility allows for a diverse and enriching special issue covering a wide range of topics, scopes and target species. The increasing accessibility of specialized equipment in tropical countries has sparked significant interest among researchers, further promoting the use of bioacoustics as a powerful tool for monitoring animal communities across large spatial and temporal scales. This issue seeks to integrate and highlight rigorous, conservation-relevant research across the tropical region, potentially establishing itself as a reference edition for a broader community interested in acoustic monitoring.

The future of bioacoustics depends not only on technological innovation, but also on building inclusive, collaborative networks and platforms that support practitioners across diverse backgrounds and levels of expertise. By reducing barriers, sharing resources and fostering global networks, the field can grow into a truly transformative force for science, conservation and equity.

## Data Availability

This article has no additional data.
